# Effects of etomidate on complications related to intubation and on mortality in septic shock patients treated with hydrocortisone: a propensity score analysis

**DOI:** 10.1186/cc11871

**Published:** 2012-11-21

**Authors:** Boris Jung, Noemie Clavieras, Stephanie Nougaret, Nicolas Molinari, Antoine Roquilly, Moussa Cisse, Julie Carr, Gerald Chanques, Karim Asehnoune, Samir Jaber

**Affiliations:** 1 Intensive Care Unit, Department of Anaesthesia and Critical Care, Saint Eloi Teaching Hospital and Institut National de la Santé et de la Recherche Médicale Unit 1046 (INSERM U-1046), Montpellier 1 University, 80 avenue Augustin Fliche, F-34295 Montpellier, Cedex 5, France; 2 Department of Abdominal Imaging, Saint Eloi Teaching Hospital, Montpellier 1 University, 80 avenue Augustin Fliche, F-34295 Montpellier, Cedex 5, France; 3 Department of Medical Statistics, Arnaud de Villeneuve Teaching Hospital, Montpellier 1 University, 80 avenue Augustin Fliche, F-34295 Montpellier, Cedex 5, France; 4 Department of Anesthesiology and Intensive Care Medicine, Hôtel Dieu Teaching Hospital, Nantes University, 1 place Alexis-Ricordeau, 44093 Nantes cedex 1, France

## Abstract

**Introduction:**

Endotracheal intubation in the ICU is associated with a high incidence of complications. Etomidate use is debated in septic shock because it increases the risk of critical illness-related corticosteroid insufficiency, which may impact outcome. We hypothesized that hydrocortisone, administered in all septic shock cases in our ICU, may counteract some negative effects of etomidate.

The aim of our study was to compare septic shock patients who received etomidate versus another induction drug both for short-term safety and for long-term outcomes.

**Methods:**

A single-center observational study was carried out in septic shock patients, treated with hydrocortisone and intubated within the first 48 hours of septic shock. Co-primary end points were life-threatening complications incidence occurring within the first hour after intubation and mortality during the ICU stay. Statistical analyses included unmatched and matched cohorts using a propensity score analysis. *P *< 0.05 was considered significant.

**Results:**

Sixty patients in the etomidate cohort and 42 patients in the non-etomidate cohort were included. Critical illness-related corticosteroid insufficiency was 79% in the etomidate cohort and 52% in the non-etomidate cohort (*P *= 0.01). After intubation, life-threatening complications occurred in 36% of the patients whatever the cohort. After adjustment with propensity score analysis, etomidate was a protective factor for death in the ICU both in unmatched (hazard ratio, 0.33 (0.15 to 0.75); *P *< 0.01)) and matched cohorts (hazard ratio, 0.33 (0.112 to 0.988); *P *= 0.04).

**Conclusion:**

In septic shock patients treated with hydrocortisone, etomidate did not decrease life-threatening complications following intubation, but when associated with hydrocortisone it also did not impair outcome.

## Introduction

Endotracheal intubation, one of the most commonly performed procedures in the ICU [1-3], is associated with a high incidence of early onset life-threatening complications (25 to 39%) because of the precarious hemodynamic and respiratory status of those patients [1,2,4]. To limit intubation-related life-threatening complications, bundle therapy including hemodynamically well-tolerated anesthetics such as etomidate has been suggested in the ICU [1,5] and is widely used in prehospital or emergency room environments [6,7]. In critically ill patients, the use of etomidate has been challenged because it inhibits adrenocortical steroid synthesis by reversibly blocking the 11β-hydroxylase enzyme action [8-10] for at least 24 hours after a single bolus [9,11]. This inhibition is associated with a risk of reversible failure of the adrenal axis, which can lead to critical illness-related corticosteroid insufficiency (CIRCI) [12]. Because CIRCI is associated with an increased mortality in septic shock patients [8,13-15], etomidate use is controversial in this setting [16-19]. Moreover, some studies suggest a link between etomidate and poor outcome [11,13,14,20-22] but others failed to confirm this link [6,23-25].

To limit the potential consequences of etomidate on the adrenal axis, hydrocortisone administration may be of interest. To our knowledge, only one randomized controlled clinical trial, performed in nonseptic critically ill patients, failed to demonstrate any benefit to counteract etomidate's side effect using a short course (48 hours) of hydrocortisone treatment [10]. In our ICU, the anesthesia bundle for intubation strongly recommends the use of a rapid sequence induction [5] and our septic shock bundle therapy includes hydrocortisone for all septic shock patients after a cosyntropin test as is frequently observed and suggested in France [15,26,27]. In our operating room, no local bundle is purposed, although ketamine and etomidate are suggested for critically ill patients. Because of its potential protective effect on intubation safety [3-5,7], due to its cardiovascular properties, and its deleterious impact on adrenal gland physiology [8,28], etomidate may have contrasting impact on the incidence of life-threatening complications occurring within 1 hour after intubation and on the long-term outcome in septic shock patients.

The present study was aimed at assessing the short-term safety and the long-term outcomes of septic patients treated with etomidate versus another induction drug for intubation. We designed the present propensity-score-driven study to evaluate, in septic shock patients, first the incidence of immediate life-threatening complications after intubation and second the long-term outcome according to the hypnotic used. The propensity score allowed us to match patients according to their probably to receive etomidate or not and to adjust for confounding factors in the present observational study.

## Materials and methods

### Study setting and patients

A cohort, observational study was performed in an adult ICU of a university hospital from June 2006 until December 2009. Data were extracted from prospective studies conducted in our ICU and previous databases [1,2,5,15,26]. The study was approved by the local ethics committee (Comité de Gestion et d'Organisation de l'Anesthésie Réanimation, Montpellier University Hospital) and, in accordance with French law, informed consent was waived. We adhered to the Strengthening the Reporting of Observational Studies in Epidemiology guidelines [29].

Etomidate blocks cortisol synthesis primarily by inhibiting the activity of 11β-hydroxylase for at least 24 to 48 hours [10,30]. Therefore, to describe the potential impact of etomidate on early and late outcome in septic shock patients, consecutive patients were eligible if they had received an induction agent for endotracheal intubation within the first 48 hours of septic shock onset. Patients were treated according to international guidelines for management of severe sepsis and septic shock [31] but all received hydrocortisone [15]. Exclusion criteria included pregnancy, age < 18 years, moribund patients, immunosuppression, and long-term or short-term corticosteroid treatment within the past 4 weeks. A cosyntropin stimulation test with 250 μg cosyntropin was performed in all septic shock patients. A 50 mg intravenous bolus of hydrocortisone was then administered every 6 hours, beginning within the first 12 hours of septic shock, for at least 5 days, tapered and stopped in 5 days according to the reversal of shock. Patients were grouped as those having received etomidate for intubation (etomidate cohort) versus those subjects having received another hypnotic (non-etomidate cohort).

### Definitions

Septic shock was defined by evidence of infection and a systemic response to infection, in addition to systolic blood pressure < 90 mmHg, despite adequate fluid replacement, or a need for vasopressors for at least 1 hour, according to the American College of Chest Physicians/Society of Critical Care Medicine Consensus Conference Committee criteria [32]. Nonresponse to the cosyntropin stimulation test using an immunoradiology assay (SP2100; Beckmancoulter SAS, Roissy, France) was defined by a delta cortisol (60 minutes after 250 μg cosyntropin) < 9 μg/dl [15,26,28]. CIRCI was defined by a delta cortisol (60 minutes after 250 μg cosyntropin) < 9 μg/dl or a baseline plasma cortisol level < 10 μg/l [12].

### Data collection

A standardized data collection instrument and guidance tool was developed for data collection. Record review and data extraction were performed by a single investigator (NC) and regular meetings were conducted to address any problems encountered during the data collection phase according to the recommendations that have been published to minimize validity threats in chart review studies [33]. Upon ICU admission, the baseline characteristics and the main variables obtained before intubation were recorded either by a nurse (from June 2006 to Jan 2009) or by computer-driven software plugged to the monitor, which recorded automatically all the variables.

At the time of intubation, clinical data including reason for intubation, interventions including sedative agent used, need for and doses of vasopressors were recorded. During the intubation procedure, drug administration and the difficulty to intubate rate (defined by three or more attempts at laryngoscopy to place the endotracheal tube into the trachea and/or > 10 minutes using conventional laryngoscopy and/or the need for another operator) [5] were documented. Within the first hour after intubation we recorded the short-term life-threatening complications that occurred, defined as previously reported [2,5]: cardiac arrest, severe cardiovascular collapse (defined as systolic blood pressure < 65 mmHg recorded at least once and/or < 90 mmHg that lasted 30 minutes despite 500 to 1,000 ml fluid loading and/or requiring introduction of vasoactive support) and severe hypoxia (defined as a decrease in SpO_2 _level < 80% during attempts). Patients who already presented a cardiovascular collapse after fluid loading or who were severely hypoxemic (SpO_2 _< 80%) after preoxygenation by noninvasive positive-pressure ventilation were not considered to have had an intubation-related complication, but rather to have presented a life-threatening condition requiring an emergency endotracheal intubation.

During the ICU stay, we documented the results for basal plasma cortisol and that after the cosyntropin test, as well as total amounts and durations of hydrocortisone and vasopressor treatments from day 0 to day 5. Outcome data include the duration of shock, length of mechanical ventilation, nosocomial infection incidence, ICU and hospital lengths of stay, and day-28 mortality.

### Statistical analysis

We had sufficient resources to review 102 patients in total. Descriptive data of quantitative variables were summarized as the mean ± standard deviation or median with interquartile range, according to the normality of the distribution, assessed with the Shapiro-Wilk test and compared with the Mann-Whitney or *t *test. Categorical data were expressed as the number and percentage and were compared with a chi-square analysis.

Using two statistical methods, we assessed the occurrence of short-term life-threatening complications and the long-term outcomes according to the administration of etomidate versus another hypnotic drug. First, unadjusted differences between patients receiving etomidate or not were compared using logistic regression after calibration with the Hosmer-Lemeshow wellness-of-fit test. Furthermore, long-term survival was assessed by a Cox regression in which we included all variables associated with *P *< 0.20 in the univariate analysis. A stepwise procedure then allowed the final multivariate model to be obtained.

Second, since patients were not randomly assigned to etomidate or other hypnotic in this observational study, we developed a propensity score using all variables associated with *P *< 0.20 in the univariate analysis. The propensity score is defined as a subject's probability of receiving a specific treatment (for example, etomidate) conditional on the observed covariates, and thus controls for selection bias in observational studies [34]. For the coupling process, optimal one-to-one nearest neighbor matching was used. When needed, patients already matched were replaced by the closest one in the in the propensity score. *P *< 0.05 was considered significant. Statistical analysis was performed by an independent statistician (NM), with R software (version 2.10.1).

## Results

### Population characteristics

During the study period, among 1,632 patients admitted to the ICU, 331 presented septic shock during their stay. Among these 331 patients, 229 either developed septic shock > 48 hours after intubation, did not have a cosyntropin test or data could not be extracted from the charts. Thus, 102 patients meeting the inclusion criteria were analyzed; 60 in the etomidate cohort and 42 in the non-etomidate cohort. The hypnotics used to induce anesthesia for intubation in the non-etomidate cohort were ketamine (*n *= 18), propofol (*n *= 10), thiopental (*n *= 13) or none (*n *= 1). The nonabdominal source of sepsis, higher Simplified Acute Physiology Score II [35] and Sequential Organ Failure Assessment [36] severity scores were more frequently observed in the etomidate cohort (Table 1).

**Table 1:** Baseline characteristics of the 102 studied patients

Characteristic	All patients (*n *= 102)	Etomidate cohort (*n *= 60)	Non-etomidate cohort (*n *= 42)	*P *value
Age (years)	69 (58 to 75)	71 (62 to 72)	68 (56 to 73)	0.18
Male gender	72 (71)	44 (73)	28 (67)	0.002
Body mass index (kg/m^2^)	25 (23 to 30)	25 (23 to 29)	26 (24 to 32)	0.31
SAPS II upon ICU admission	48 (40 to 63)	52 (42 to 65)	46 (34 to 58)	0.049
SOFA score upon ICU admission	8 (6 to 12)	10 (7 to 13)	8 (6 to 11)	0.04
Previous disease				
Hypertension	43 (42)	26 (43)	17 (41)	0.77
Coronary artery disease	22 (22)	14 (23)	8 (19)	0.04
Congestive heart failure	29 (28)	17 (27)	12 (29)	0.98
Neurological disease	20 (20)	11 (18)	9 (21)	0.70
Chronic obstructive pulmonary disease	18 (18)	10 (17)	8 (19)	0.76
Diabetes mellitus	20 (20)	10 (17)	10 (24)	0.37
Cancer	41 (40)	26 (43)	15 (36)	0.44
Liver cirrhosis	22 (21)	14 (23)	8 (19)	0.61
Admitting diagnosis group				
Medical	42 (41)	27 (45)	15 (36)	0.35
Emergency surgery	45 (44)	23 (38)	22 (52)	0.16
Elective surgery	15 (15)	10 (17)	5 (12)	0.55
Time from infection diagnostic to surgery (hours)	8 (4 to 24)	8 (5 to 24)	8 (4 to 24)	> 0.99
Source of sepsis				
Pulmonary	33 (32)	21 (35)	12 (29)	0.49
Abdominal	54 (53)	25 (42)	29 (69)	0.02
Other	15 (15)	14 (23)	1 (2)	0.03
Appropriateness of initial antibiotic therapy	68/89 (76)	39/52 (75)	29/37 (78)	0.71
Main variables obtained before intubation				
Systolic blood pressure < 90 mmHg	22(39)	15 (45)	7 (29)	0.31
SpO_2 _below 80%	6 (10)	5 (15)	1 (4)	0.21
Lactatemia (mmol/l)	2.5 (1.1 to 4.7)	2.5 (1.1 to 5.3)	2.3 (1.4 to 4.1)	0.80
Vasopressors use	21 (21)	16 (27)	5 (12)	0.09
Myorelaxant use to facilitate intubation	97 (98)	57 (95)	40 (98)	0.96

Data presented as number (%) or median (quartiles). Appropriateness of initial antibiotic therapy was expressed as the number of appropriate first-line antibiotic therapies over the number of charts that were exploitable. SAPS II, Simplified Acute Physiology Score II [35]; SOFA, Sequential Organ Failure Assessment [36].

We first evaluated the association of hypnotics, intubation-related life-threatening complications and outcome in unmatched cohorts.

### Intubation procedure and intubation-related complications

**Figure 1:**
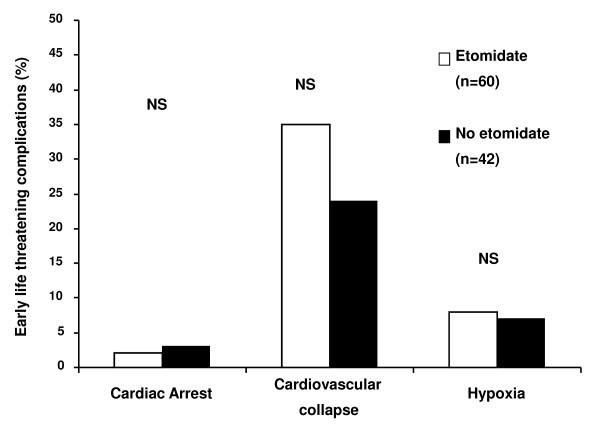
**Incidence of life-threatening complications according to the hypnotic used to facilitate intubation**. No difference in life threatening complications rates was found between the hypnotic used. NS, not significant.

Intubation was indicated mainly for urgent surgery (42%) or acute respiratory failure (35%). Myorelaxants were used in nearly all of the procedures without any complications related to their use (Table 1). Intubation was difficult in 10 cases (10%), independent of the administered hypnotic. Short-term life-threatening complications within 1 hour of intubation occurred in 37 (36%) of the 102 studied patients (Figure 1). In univariate analysis, the Simplified Acute Physiology Score II was associated with a higher risk of complications and both the administration of norepinephrine prior to intubation and the use of a drug other than etomidate to facilitate intubation were associated with a lower risk of complications (Table 2). In multivariate analysis, the administration of norepinephrine prior to intubation was the sole independent protective factor for life-threatening complications occurring after intubation (Table 2).

**Table 2:** Comparison of main variables obtained before intubation according to occurrence of a short-term life-threatening complication

	Univariate analysis			Multivariate analysis	
	
	No life-threatening complications following intubation (*n *= 65)	Life-threatening complications following intubation (*n *= 37)	*P *value	Odds ratio (95% CI)	*P *value
SAPS II upon ICU admission	48 (37 to 59)	54 (44 to 70)	< 0.01	1.04 (0.99 to 1.08)	0.08
SOFA score upon ICU admission	8 (6 to 12)	8 (6 to 11)	0.75		
Main variables obtained before intubation					
Lactatemia (mmol/l)	2.6 (1.1 to 4.9)	2.2 (1.5 to 3.9)	0.89		
Vasopressors use	19 (29)	2 (6)	< 0.01	0.11 (0.01 to 0.93)	0.04
Lowest systolic blood pressure recorded within 30 minutes before intubation (mmHg)	89 (80 to 120)	100 (90 to 122)	0.08	1.01 (0.99 to 1.03)	0.09
Drug used to facilitate intubation					
Etomidate	35 (53)	25 (69)	0.18		
Other	31 (47)	11 (31)	0.05	0.60 (0.18 to 2.03)	0.41
Myorelaxants	62 (95)	35 (97)	0.86		

Data presented as number (%) or median (quartiles). Multivariate analysis was performed using a logistic regression, which assesses the risk of life-threatening complication within the first hour after intubation. Each variable with *P *< 0.20 in the univariate analysis was entered in the model. Lowest systolic blood pressure before intubation, according to its median value, was forced into this model. CI, confidence interval; SAPS II, Simplified Acute Physiology Score II [35]; SOFA, Sequential Organ Failure Assessment [36].

### Critical illness-related corticosteroid insufficiency and hydrocortisone treatment

Patients were compared according to the hypnotic they received to facilitate intubation. The cosyntropin test was performed within 24 hours after intubation in 85% of the patients, and after the first 24 hours in 15% of the population but always before the first dose of hydrocortisone. Hydrocortisone treatment was started 540 (300 to 1,125) minutes after intubation. The basal plasma cortisol concentration was significantly lower (19 (14 to 35) μg/dl versus 31 (17 to 45) μg/dl; *P *= 0.04) and the percentage of nonresponders to the cosyntropin stimulation test was significantly higher (79% vs. 52%; *P *= 0.01) in the etomidate cohort compared with the non-etomidate cohort. CIRCI was also significantly more frequently observed in the etomidate cohort compared with the non-etomidate cohort (79% vs. 59%; *P *= 0.04). In the etomidate cohort, the cumulative hydrocortisone dose was significantly higher (1,250 (650 to 1,650) mg vs. 750 (350 to 1,150) mg; *P *= 0.02) and the duration of treatment was significantly longer (168 (96 to 216) hours vs. 96 (48 to 162) hours; *P *= 0.01) than in the non-etomidate cohort.

### Reversal of shock

Norepinephrine was administered within 12 hours after intubation in 100% of the patients without significant difference between cohorts. Patients in the etomidate cohort needed a higher cumulative dose of norepinephrine during their ICU stay compared with patients anesthetized with another hypnotic (95 (39 to 203) mg vs. 58 (30 to 97) mg from day 0 to day 5; *P *= 0.02). The duration of norepinephrine treatment was not different between cohorts (58 (37 to 94) hours in the etomidate cohort vs. 48 (25 to 81) hours in the non-etomidate cohort; *P *= 0.20).

### ICU length of stay and complications

The incidence of nosocomial infections, length of mechanical ventilation, and lengths of ICU and hospital stay did not significantly differ between cohorts (Table 3).

**Table 3:** Long-term outcome according to the hypnotic used to facilitate intubation

	Etomidate cohort (*n *= 60)	Non-etomidate cohort (*n *= 42)	*P *value
Number of nosocomial infections	38 (100)	22 (100)	0.84
Pneumonia	20 (53)	10 (45)	0.30
Urinary tract infections	10 (26)	7 (32)	> 0.99
Central venous catheter-related infections	8 (21)	5 (23)	0.81
Length of mechanical ventilation (days)	5 (2 to 14.8)	5 (1 to 7)	0.10
ICU length of stay (days)	12 (6 to 22)	9 (4 to 13)	0.06
Hospital length of stay (days)	32 (22 to 50)	29 (19 to 45)	0.18
Mortality at day 28	17 (28)	14 (33)	0.59

Data presented as number (%) or median (quartiles). Nosocomial infections are expressed as the total number during the ICU stay and results are expressed as the percentage of total nosocomial infections that came out during the ICU stay. Patients could develop more than one infection during the ICU stay.

### Mortality

**Figure 2:**
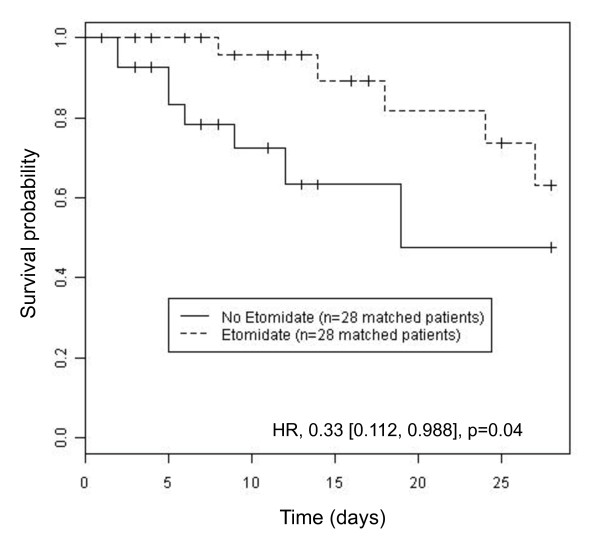
**Kaplan-Meier curves comparing survival probability after propensity score adjustment in etomidate and non-etomidate cohorts**. Etomidate was associated with a significant lower risk of mortality at day 28. HR, hazard ratio.

Although the crude day-28 mortality was not different according to the drug used to facilitate intubation, the Cox regression model yielded a hazard ratio for death at day 28 in the etomidate cohort, as compared with the non-etomidate cohort, of 0.33 (0.12 to 0.90; *P *= 0.03) (Table 4). The Hosmer-Lemeshow test showed that the model fits to predict mortality, with 82% of well-classed patients and *P *= 0.16. Second, we evaluated the association of hypnotics and both intubation-related life-threatening complications and outcome in matched cohorts. Propensity score matching resulted in a cohort of 56 patients, with 28 patients who received etomidate and 28 patients who did not receive etomidate. In this cohort of 56 patients, matching was based on etomidate use, the Simplified Acute Physiology Score II score without counting age and the basal plasma cortisol level. The occurrence of intubation-related life-threatening complications was similar in both the etomidate and the non-etomidate cohorts. The Kaplan-Meier estimator for 28-day mortality using propensity score matching was significantly lower in the etomidate cohort than in the non-etomidate cohort and showed a hazard ratio for death in the ICU in the etomidate cohort, as compared with the non-etomidate cohort, of 0.33 (0.112 to 0.988) (Figure 2). The *c*-statistic for the propensity score was 0.7794.

**Table 4:** Comparison of main variables before intubation and cosyntropin test results between day-28 survivors and nonsurvivors

				Multivariate analysis	
				
	Survivors (*n *= 66)	Nonsurvivors (*n *= 36)	*P *value	Hazard ratio (95% CI)	*P *value
SAPS II upon ICU admission	45 (37 to 55)	60 (47 to 71)	< 0.01	1.04 (1.01 to 1.06)	< 0.01
SOFA score upon ICU admission	8 (5 to 11)	11 (8 to 13)	0.08	1.01 (0.89 to 1.16)	0.85
Main variables obtained before intubation					
Vasopressor use	10 (15)	11 (31)	> 0.99		
Drug used to facilitate intubation					
Etomidate	41 (62)	19 (53)	0.17	0.33 (0.12 to 0.90)	0.03
Other	25 (38)	17 (47)	0.81		
Myorelaxants	62 (94)	35 (97)	0.46		
Basal cortisol plasma level (μg/dl)	20 (14 to 40)	33 (19 to 49)	0.08	0.99 (0.96 to 1.03)	0.85
Cortisol plasma level after ACTH test (μg/dl)	31 (18 to 44)	35 (21 to 48)	0.54		
Cosyntropin test responders	21/59 (36)	4/22 (19)	0.13		

Data presented as number (%) or median (quartiles). Multivariate analysis was performed using a Cox regression analysis for mortality. All variables with *P *< 0.20 in the univariate analysis were entered in the model. ACTH, Adrenocorticotropic hormone; CI, confidence interval; SAPS II, Simplified Acute Physiology Score II [35]; 95% CI: 95% confidence interval; SOFA, Sequential Organ Failure Assessment [36].

## Discussion

The results of our study show that, first, intubation in septic shock patients was associated with a 36% rate of short-term life-threatening complications and that this rate was independent of the hypnotic used to facilitate the procedure. Second, to our surprise, in unmatched cohorts and after matching using a propensity score analysis, the administration of a single dose of etomidate in septic shock patients treated with hydrocortisone was associated with a lower risk of day-28 mortality (Table 3).

Potential confounding factors of the study must be addressed. First, this study was a single-center observational study in which the hypnotic used for induction of anesthesia was not randomized. Second, this was a small study subject to unmeasured or residual confounding (for example, patient heterogeneity, heterogeneity for intubation indication, protocol deviation), which is a limitation. The propensity score, however, is a tool to increase the accuracy of results in cohort studies [37,38]. Moreover, external validity of observational studies may be higher than for randomized controlled trials. Third, because of the study design, we cannot provide detailed explanations about the protective mechanisms of etomidate on long-term outcomes.

In the present study, the hypnotic used to facilitate intubation in critically ill patients was mainly etomidate to limit the risk of cardiovascular collapse that may occur after intubation [5]. Propofol or pentobarbital represented 20% of the administered hypnotics (Table 2), mainly in the operating room for urgent surgery. The difficult intubation rate was high (near 10%), which is above the usual rate in the operating room but is similar to the rate reported in the few studies existing in this field [2,5]. To facilitate intubation, almost all of the patients received a myorelaxant agent (Table 1), mostly succinylcholine, as recommended by our local protocol. Interestingly, the short-term life-threatening complications that occurred within 1 hour after intubation concerned 36% of the patients. This rate is similar to that in the literature [2,4] and above the rate we reported after the implementation of a care bundle in nonselected critically ill patients [5]. The discrepancy between the present study and our previous results [5] may be explained by the severity of the patients in the present study, all of them intubated with cardiovascular instability related to sepsis. In the multivariate analysis, the sole factor associated with short-term outcome was the administration, prior to intubation, of norepinephrine (Table 2). Norepinephrine administration before intubation may be protective by both limiting the risk of severe cardiovascular collapse following sympatholysis induced by the hypnotic and the detrimental effect of thoracic positive pressure on venous return. In our unit, norepinephrine prior to induction is suggested for diastolic blood pressure < 45 to 50 mmHg [5].

In the present study, we assessed the short-term life-threatening complication rate, but also the long-term effect of hypnotics on outcome. Patients intubated with etomidate were more likely to present CIRCI (Table 4) and needed a longer hydrocortisone treatment and a higher total amount of hydrocortisone. One bolus of etomidate impairs cortisol secretion [8,9,39,40] by the inhibition, for at least 24 to 48 hours, of 11β-hydroxylase, the enzyme that converts 11β-deoxycortisol to cortisol in critically ill patients [8,10,21]. The higher rate of CIRCI when patients received etomidate may explain the higher cumulative dose of hydrocortisone because, in the present study, hydrocortisone was tapered and stopped according to the reversal of shock. CIRCI is associated with increased morbidity and mortality in septic shock patients [8,13,14,22]. However, despite a higher rate of CIRCI, we showed that etomidate was a protective factor for mortality in both unmatched and matched cohorts (Figure 2).

Our study provides new data on the effect of etomidate in septic shock. In a *post-hoc *analysis of a multiple-center trial designed to evaluate the impact of hydrocortisone treatment in septic shock patients, the authors reported an increased death rate in patients that had been intubated with etomidate compared with other hypnotics [28]. In contradiction, this increase was not statistically significant after adjustment in a multivariate analysis [21]. Furthermore, Cuthbertson and colleagues showed that administration of etomidate was associated with increased mortality, but in only one of two multiple regression models [20]. Despite higher severity of illness scores in patients intubated with etomidate compared with patients intubated with another hypnotic (Table 1), our study demonstrated a protective effect of etomidate on day-28 mortality using Cox regression. This effect was confirmed after matching (Figure 2).

The consequences of etomidate on long-term outcomes in the present study must be discussed in light of the co-administration of hydrocortisone. In the present study, hydrocortisone treatment was started within the first 12 hours after etomidate administration, earlier than in other studies [28]. To date, studies have failed to demonstrate an improved outcome when supplementing etomidate treatment with corticosteroids [10,22,24] and hydrocortisone is not recommended in every patient presenting septic shock but is suggested in those refractory to fluid challenge and dependent on high-dose vasopressors [12]. However, because the inhibition of cortisol synthesis due to etomidate is immediate, hydrocortisone must be administered immediately after an etomidate bolus to counter its effects on steroid synthesis [20]. Evaluating the role of hydrocortisone in patients who received etomidate may thus be interesting. To explain the impact of etomidate, it has also been reported that ketamine - which was the main drug used in the non-etomidate cohort - may have an anti-inflammatory effect in experimental sepsis models [41,42]. Whether this anti-inflammatory effect may exacerbate late sepsis-induced immunosuppression, however, is unknown.

## Conclusion

We have reported that etomidate use for intubation in septic shock patients treated with hydrocortisone did not prevent short-term life-threatening complications following intubation despite its cardiovascular tolerance profile. Our study also suggests that patients co-treated with etomidate and hydrocortisone might not be associated with a worse outcome than another hypnotic used to facilitate intubation. Future randomized controlled studies should be performed to confirm this result and to evaluate early hydrocortisone treatment in septic shock patients who received etomidate.

## Key messages

• In septic shock patients treated with hydrocortisone, despite its cardiovascular tolerance, etomidate was not associated with a decrease of life-threatening complications following intubation in comparison with other hypnotics.

• Etomidate was associated with a longer period of shock and higher cumulative dose of hydrocortisone than patients intubated with another hypnotic.

• Interestingly, patients treated with etomidate and hydrocortisone presented a lower risk of day-28 mortality, both in unmatched and matched cohorts and multivariate analysis.

## Abbreviations

CIRCI: critical illness-related corticosteroid insufficiency.

## Competing interests

BJ received funding from Merck, but not in relation to the present study. KA received honoraria from B-Braun Medical, Fresenius, and LFB for public speaking, but not in relation to the present study. SJ received honoraria from Maquet, Draeger, Hamilton Medical, Fisher Paykel, and Abbott, but not in relation to the present study. The remaining authors declare that they have no competing interests.

## Authors' contributions

BJ, NC and SJ designed the study protocol and wrote the report. BJ, SN and NM were responsible for statistical analyses. NC performed the chart review. AR, GC and KA made substantial modifications to the report. SN, JC and MC helped with the report's correction and chart review. All authors read and approved the manuscript for publication.
